# Cardiac Structural and Functional Remodeling After Transcatheter Mitral Valve in Valve Implantation: Early Changes and Prognostic Significance

**DOI:** 10.1016/j.shj.2023.100264

**Published:** 2023-12-26

**Authors:** Gloria Ayuba, Zhiying Meng, Abigail S. Baldridge, Ansh Goyal, Blair Tilkens, Rishi Shrivastav, Taimur Safder, Chris S. Malaisrie, James Flaherty, Patrick M. McCarthy, James D. Thomas, Charles Davidson, Jyothy Puthumana, Akhil Narang

**Affiliations:** a Division of Cardiology, Department of Medicine, Northwestern University Feinberg School of Medicine, Bluhm Cardiovascular Institute, Chicago, Illinois; b Division of Cardiology, Department of Medicine, University of Texas Southwestern, Dallas, Texas; c Department of Cardiac Surgery, Northwestern University Feinberg School of Medicine, Bluhm Cardiovascular Institute Chicago, Illinois

**Keywords:** Left atrial remodeling, Left atrial strain, Prosthetic valve failure, Right ventricular free wall strain, Transcatheter mitral valve-in-valve

## Abstract

**Background:**

Transcatheter mitral valve-in-valve (MViV) replacement has emerged as an alternative to redo mitral valve (MV) surgery for the management of failed bioprosthetic MVs. The degree of cardiac remodeling assessed by echocardiography has been shown to have prognostic implications in degenerative mitral regurgitation patients undergoing MV surgery. The impact of transcatheter MViV in patients with degenerative bioprosthetic MV failure on cardiac remodeling and its associated prognosis remains undescribed.

**Objectives:**

The aim of this study is to describe the early anatomic and functional changes of the left-sided chambers and right ventricle by echocardiography posttranscatheter MViV intervention and their impact on mortality outcomes. Additionally, we sought to analyze the outcome of heart failure in bioprosthetic MV failure patients undergoing transcatheter MViV replacement.

**Methods:**

We analyzed consecutive patients undergoing MViV intervention for symptomatic bioprosthetic MV failure. Echocardiograms before intervention and within 100 days postintervention were analyzed. A chart review was performed to obtain baseline characteristics, follow-up visits, 30-day heart failure and 1-year all-cause mortality outcomes.

**Results:**

A total of 62 patients (mean age 69 ± 13 years, 61% male) were included in the study. Most patients were undergoing MViV intervention for prosthetic mitral stenosis n = 48 (77.4%) and the rest for mitral regurgitation or mixed disease. Compared with baseline, significant reductions were observed in median left atrial volume (LAV; 103 [81–129] ml vs. 95.2 [74.5–117.5] ml, *p* < 0.01) and mean (SD) left atrial conduit strain (9.1% ± 5.2% vs. 10.8% ± 4.8%, *p* = 0.039) within 100 days postintervention. Early reduction in right ventricular free wall global longitudinal strain and fractional area change also occurred postintervention. No significant change in left ventricular chamber dimensions or ejection fraction was observed. During the 1-year follow up period, 5 (8%) patients died. While baseline LAV was not associated with 1-year all-cause mortality (OR 0.98 CI 0.95–1.01; *p* = 0.27), a change in LAV in the follow up period was associated with all-cause mortality at 1 year (OR 1.06 CI 1.01–1.12; *p* = 0.023). At 30 days postintervention, 65% of patients had an improvement in their New York Heart Association functional class.

**Conclusion:**

In this retrospective study of patients undergoing transcatheter MViV intervention for failed bioprosthetic MVs, early reverse remodeling of the left atrium occurs within 100 days postintervention and reduction in LAV is associated with reduced all-cause mortality at 1 year. In addition, there is significant improvement in heart failure symptoms at 30 days following intervention but further investigation into the longitudinal remodeling changes and long-term outcomes is needed.

## Introduction

Bioprosthetic mitral valve (MV) degeneration that requires repeat intervention within 10 years postimplantation occurs in up to 35% of patients. MViV has emerged as an alternative to redo MV surgery for the management of these high surgical risk patients.^1^, ^2^, ^3^, ^4^

The procedural success and durability of transcatheter MV replacement has been reported in outcome studies from the Society of Thoracic Surgeons/American College of Cardiology Transcatheter Valve Therapy Registry (TVT), with higher procedural success and lower short-term mortality reported in MV-in-valve (MViV) intervention cohorts compared to valve-in-ring and valve-in-mitral annular calcification cohorts. Those undergoing MViV intervention showed sustained clinical improvement in heart failure with an overall all-cause mortality of approximately 5% at 30 days and 17% at 1 year.^5^^,^^6^

This success is likely driven by underlying anatomical and functional changes in heart chamber dynamics that are not yet completely understood. Changes in chamber size are a hallmark of structural remodeling due to changes in the function of the chambers. Cardiac functional remodeling can be assessed by evaluating for atrial and ventricular strain using two-dimensional speckle-tracking echocardiography and has been proven to be feasible and reproducible.^7^, ^8^, ^9^, ^10^, ^11^, ^12^, ^13^

Left atrial strain reflects the role of the left atrium in modulating left ventricular filling in different phases of the cardiac cycle. Peak atrial longitudinal strain (PALS) contributes about 40%-50% of total LA function and reflects the end of the reservoir phase when the LA fills and reaches its peak in systole. Following this, passive left atrial emptying, also known as the conduit phase, can be captured by conduit strain (CS) measurements and contributes to about 25% of atrial function. Finally, atrial contraction which can be measured as the peak atrial contractile strain contributes to about 25% of atrial function.^7^^,^^14^ Similarly, right ventricular remodeling and function can be understood through dimensions and pressures at various phases, as well as through echocardiographic measurements of tricuspid annular plane systolic excursion (TAPSE), lateral tricuspid annulus peak systolic velocity (S′), fractional area change (FAC), and strain.^15^^,^^16^

In short, though the effect of MV surgery on left atrial and biventricular remodeling and prognosis has been extensively studied,^8^^,^^10^, ^11^, ^12^, ^13^^,^^17^, ^18^, ^19^ there remains a paucity of data on the effect of MViV replacement on cardiac structural and functional remodeling and the prognostic impact of changes in chamber dimensions, function, and reverse cardiac remodeling in patients with degenerative bioprosthetic MV failure.

Therefore, the aim of this study was to describe the early anatomic and functional changes of the left-sided chambers and right ventricle by echocardiography due to transcatheter MViV intervention and to determine their association with the primary outcome of all-cause mortality at 1 year. Additionally, we sought to analyze the impact of MViV replacement on heart failure symptom improvement.

## Methods

This study was based on the experience at Northwestern University with transcatheter MViV replacement between January 2016 and October 2021. Patients were identified and data was collected from comprehensive 2-dimensional echocardiograms performed before intervention (baseline) and within 100 days postintervention (follow-up). Outcome data for all patients undergoing percutaneous transcatheter MViV replacement with balloon-expandable SAPIEN 3 (Edwards Lifesciences, Irvine, California) transcatheter heart valves via transeptal approach^20^ for symptomatic bioprosthetic MV failure was obtained from the TVT registry. The data were collected by trained abstractors who are unaffiliated with the author team.

The study protocol was approved and granted a waiver of informed consent by the Northwestern University institutional review board.

### Echocardiographic Analysis

All two-dimensional echocardiograms included in the study were analyzed by an advanced cardiovascular imaging trained cardiologist following standardized American Society of Echocardiography guidelines^21^^,^^22^ with assessment of variables including left ventricular ejection fraction by biplane Simpson's method of disks, left ventricular end systolic dimension, left ventricular end diastolic dimension, left atrial volume (LAV) by biplane method, right ventricular fractional area change, TAPSE, right ventricular peak systolic velocity, and MV gradient. All strain assessment was performed via speckle tracking analysis using TomTec Arena 2.41 (Philips); 2D echocardiograms with inadequate images for myocardial deformation analysis (defined as inadequate quality of tracking in ≥2 segments) were not utilized.

### Statistical Analysis

For descriptive statistics, continuous variables were presented as mean ± standard deviation (SD) or median (IQR) as appropriate. Categorical variables were presented as frequency and percentages. Baseline characteristics were compared between patients with and without 1-year mortality using two sample *t*-test, Wilcoxon test, Chi-squared test or Fisher exact test as appropriate.

Pre- and post-MViV intervention echocardiographic parameters of interest were compared using the paired *t*-test and Wilcoxon sign-rank test as appropriate. Cox proportional hazards regression models adjusted for age and gender were used to evaluate the association between baseline echocardiogram parameters and early change in echo parameters with 1-year mortality.

A subgroup analysis was also conducted to evaluate differences of echocardiographic parameters pre- and post-MViV intervention in patients with atrial fibrillation/flutter. Comparisons between groups were performed using two sample *t*-test, or Wilcoxon test as appropriate.

Improvement in New York Heart Association (NYHA) class distribution 1 month after MViV procedure was assessed graphically using an alluvial flow plot. In exploratory analyses, echocardiographic parameters were further compared between valve lesion and postprocedural residual MR groups using Analysis of Variance or Kruskal-Wallis test.

All statistical analyses were performed using SAS (Base 9.4) software, with *p*-values of <0.05 considered statistically significant.

## Results

During the study period, 62 patients had transcatheter MViV intervention, with a mean age of 69 ± 13 years, and 61% were male (Table 1). There were no significant differences in baseline characteristics between those who survived at 1 ​year and those who did not.

**Table 1:** Baseline characteristics of 62 patients undergoing transcatheter mitral valve-in-valve implantation

Characteristic, no. (%)	Overall		Mortality at 1 y		
	N	Descriptive	No (n = 57)	Yes (n = 5)	*p* value∗
Age, years, mean (SD)	62	69.3 ± 12.5	68.9 ± 12.9	74.0 ± 9.3	0.39
Female	62	24 (38.7)	21 (36.8)	3 (60.0)	0.31
Body mass index, kg/m^2^, mean (SD)	62	27.3 ± 6.2	27.5 ± 6.4	25.2 ± 3.0	0.43
Race	62				0.27
White or Caucasian		40 (64.5)	37 (64.9)	3 (60.0)	
Black or African American		7 (11.3)	6 (10.5)	1 (20.0)	
Asian		4 (6.5)	3 (5.3)	1 (20.0)	
Other/Unknown		11 (17.7)	11 (19.3)	0 (0.0)	
Atrial fibrillation	62	33 (53.2)	31 (54.4)	2 (40.0)	0.66
Atrial flutter	62	7 (11.3)	6 (10.5)	1 (20.0)	0.46
Heart failure	62	58 (93.5)	53 (93.0)	5 (100.0)	1.00
Coronary artery disease	62	40 (64.5)	37 (64.9)	3 (60.0)	1.00
Hypertension	62	50 (80.6)	46 (80.7)	4 (80.0)	1.00
Tobacco use	62	2 (3.2)	2 (3.5)	0 (0)	1.00
Lung disease	62	15 (24.2)	13 (22.8)	2 (40.0)	0.59
Angiotensin-converting enzyme (ACE) inhibitor	62	24 (38.7)	22 (38.6)	2 (40.0)	1.00
Angiotensin II receptor blockers (ARBs)	62	12 (19.4)	10 (17.5)	2 (40.0)	0.24
Beta-blocker	62	55 (88.7)	50 (87.7)	5 (100)	1.00
Diuretics	62	58 (93.5)	53 (93.0)	5 (100)	1.00
Heart rate, bpm, median (IQR)	61	70 (61-83)	70 (61-84)	80 (70-81)	0.54
Residual mitral regurgitation	60				0.20
None		20 (33.3)	20 (35.7)	0 (0)	
Trace		31 (51.7)	27 (48.2)	4 (100)	
Mild		9 (15.0)	9 (16.1)	0 (0)	
Procedure reason	62				0.15
Mitral stenosis		48 (77.4)	44 (77.2)	4 (80.0)	
Prosthetic mitral regurgitation		9 (14.5)	9 (15.8)	0 (0)	
Mixed		5 (8.1)	4 (7.0)	1 (20.0)	
NYHA	61				0.08
Class II		10 (16.4)	10 (17.9)	0 (0)	
Class III		41 (67.2)	38 (67.9)	3 (60.0)	
Class IV		10 (16.4)	8 (14.3)	2 (40.0)	
STS risk score, median (IQR)	46	5.5 (3.6-11.5)	5.0 (3.6-9.5)	16.4 (6.5-26.1)	0.24

Abbreviations: IQR, interquartile range; NYHA, New York Heart Association.

∗ Two sample *t* test, Wilcoxon test, Chi-squared test or Fisher exact test as appropriate.

Fifty-seven (92%) patients had preintervention and follow-up (within 100 days after intervention) echocardiography that allowed measurement of at least the LAV.

Patients underwent MViV intervention for prosthetic mitral stenosis (n = 48, 77%), prosthetic mitral regurgitation (n = 9, 14%), and mixed valve disease (n = 5, 8%) with a median STS risk score of 5.5% for re-do MV replacement. Forty patients (65%) had trace to mild residual mitral regurgitation following intervention and 33% had no residual mitral regurgitation.

Atrial fibrillation/flutter was present in 40 patients (65%) and 51 patients (84%) had NYHA class III/IV heart failure at baseline.

### Early Structural and Functional Remodeling Following Transcatheter Mitral Valve-In-Valve Intervention

Compared to baseline, median LAV was reduced following intervention (103 [81-129] ml vs. 95.2 [74.5-117.5] ml, *p* < 0.01) (Table 2).

**Table 2:** Preprocedure and postprocedure echocardiographic characteristics of patients undergoing transcatheter mitral valve-in-valve implantation

Echo parameters, mean (SD)	Preprocedure		Postprocedure		*p* value∗
	N	Descriptive	N	Descriptive	
LVEF, %	57	59.5 ± 10.2	56	59.6 ± 11.9	0.81
LVESD, cm	57	3.0 ± 0.9	56	3.0 ± 0.9	0.41
LVEDD, cm	57	4.5 ± 0.9	56	4.4 ± 0.9	0.86
LVGLS, %	55	-16.4 ± 4.6	55	-15.2 ± 4.7	0.13
LAV, ml, median (IQR)	57	103.0 (81.0-129.0)	56	95.2 (74.5-117.5)	<0.01
PALS, %, median (IQR)	49	11.7 (8.0-17.3)	53	13.0 (9.9-18.2)	0.08
CS, %	49	9.1 ± 5.2	53	10.8 ± 4.8	0.039
PACS, %, median (IQR)	49	2.5 (1.1-5.7)	53	2.1 (1.0-4.7)	0.32
RVFAC, %	52	20.3 ± 9.7	56	26.8 ± 9.5	<0.01
TAPSE, cm, median (IQR)	53	1.5 (1.2-1.7)	54	1.7 (1.3-1.9)	0.11
S’, cm/s	47	8.8 ± 2.4	56	8.4 ± 2.3	0.29
RVFWS, %	51	11.2 ± 4.5	54	14.1 ± 4.9	<0.01
MV gradient, mmHg	56	13.4 ± 5.1	56	7.6 ± 3.0	<0.01
RVSP, mmHg	56	61.4 ± 16.5	55	49.2 ± 19.9	<0.01
Heart rate, bpm, median (IQR)	56	70 (61-84)	56	71 (65-83)	0.71
SBP, mmHg, median (IQR)	54	120 (104-134)	47	120 (111-135)	0.52

Abbreviations: CS, conduit strain; IQR, interquartile range; LAV, left atrial volume; LVEF, left ventricular ejection fraction; LVEDD, left ventricular end-diastolic dimension; LVESD, left ventricular end-systolic dimension; LVGLS, left ventricular global longitudinal strain; MV, mitral valve; PACS, peak atrial contractile strain; PALS, peak atrial longitudinal strain; RVSP, right ventricular systolic pressure; RVFAC, right ventricular fractional area change; RVFWS, right ventricular free-wall strain; SBP, systolic blood pressure; TAPSE, tricuspid annular plane systolic excursion.

∗ Paired *t* test, Wilcoxon sign-rank test as appropriate.

Left atrial CS (*p* = 0.039) was increased significantly after intervention. No change from baseline was found in left atrial PALS (*p* = 0.08), or peak atrial contractile strain (*p* = 0.32) after intervention (Table 2).

Assessment of the right ventricular chamber following MViV intervention showed reduction in right ventricular systolic pressure (61.4mmHg ±16.5 vs. 49.2mmHg ±19.9) (*p* < 0.01) and improvement in right ventricular function as detected by right ventricular free wall longitudinal strain (11.2% ± 4.5% vs. 14.1% ± 4.9) (*p* < 0.01) and FAC (20.3% ± 9.7% vs. 26.8% ± 9.5) (*p* < 0.01) compared to baseline (Table 2).

We additionally observed no structural or functional remodeling of the left ventricle as determined by left ventricular chamber dimension (Left ventricular end diastolic dimension; *p* = 0.86), ejection fraction (*p* = 0.81) and global longitudinal strain (*p* = 0.13) (Table 2).

Mean MV gradient was also reduced following intervention compared to baseline (MV gradient; (13.4mmHg ±5.1 vs. 7.6mmHg ±3.0) (*p* ≤ 0.01) without significant difference in heart rate (*p* = 0.71).

We observed significant differences in baseline MV gradients between valve lesion groups with higher gradients noted in patients with prosthetic stenosis (14.4mmHg ±4.6) compared to those with regurgitation (7.6mmHg ±2.7) or mixed valve disorders (11.8mmHg ±4.6) (*p* < 0.01) but no significant difference postintervention (*p* = 0.05) (Supplemental Table 1).

Although 65% of patients had trace to mild residual mitral regurgitation following intervention, no echocardiographic parameter was significantly associated with postprocedure residual mitral regurgitation (Supplemental Table 2).

### Association of Baseline Echocardiography Parameters and Early Cardiac Remodeling with 1-year all-cause Mortality

During the 1-year follow up period postintervention, 5 (8%) patients died.

All-cause mortality at 1 year was not associated with baseline echo derived left-sided chamber size or function parameters evaluated, nor with right ventricular systolic pressure or right ventricular function as assessed by FAC, TAPSE, S′ or right ventricular free wall strain (Table 3). We further evaluated the association of CS and mortality, adjusted for left ventricular ejection fraction and left ventricular global longitudinal strain, showing that the results appear to be similar compared to the model without adjustment (Supplementary Table 3).

**Table 3:** Association of baseline echo parameters and 1-year mortality of 62 patients undergoing transcatheter mitral valve-in-valve implantation

Characteristic	Hazard ratio (95% CI)∗	*p* value
LVEF, %	1.08 (0.96 - 1.22)	0.19
LVESD, cm	0.41 (0.10 - 1.70)	0.22
LVEDD, cm	0.51 (0.12 - 2.04)	0.34
LVGLS, %	1.02 (0.82 - 1.25)	0.88
LAV, ml	0.98 (0.95 - 1.01)	0.27
PALS, %	0.95 (0.81 - 1.11)	0.52
CS, %	0.90 (0.72 - 1.12)	0.35
PACS, %	1.03 (0.80 - 1.32)	0.82
RVFAC, %	0.998 (0.90 - 1.11)	0.97
TAPSE, cm	1.04 (0.81 - 1.33)	0.77
S’, cm/s	0.96 (0.66 - 1.39)	0.82
RVFWS, %	0.96 (0.78 - 1.17)	0.66
RVSP, mmHg	1.03 (0.98 - 1.09)	0.22
MV gradient, mmHg	1.01 (0.83 - 1.22)	0.95
Heart rate, bpm	1.02 (0.95 - 1.09)	0.63

Abbreviations: CI, confidence interval; CS, conduit strain; LAV, left atrial volume; LVEF, left ventricular ejection fraction; LVEDD, left ventricular end-diastolic dimension; LVESD, left ventricular end-systolic dimension; LVGLS, left ventricular global longitudinal strain; MV, mitral valve; PACS, peak atrial contractile strain; PALS, peak atrial longitudinal strain; RVSP, right ventricular systolic pressure; RVFAC, right ventricular fractional area change; RVFWS, right ventricular free-wall strain; TAPSE, tricuspid annular plane systolic excursion.

∗ Cox regression modeling hazard ratio of 1-year mortality adjusting for age and gender.

**Figure 1:**
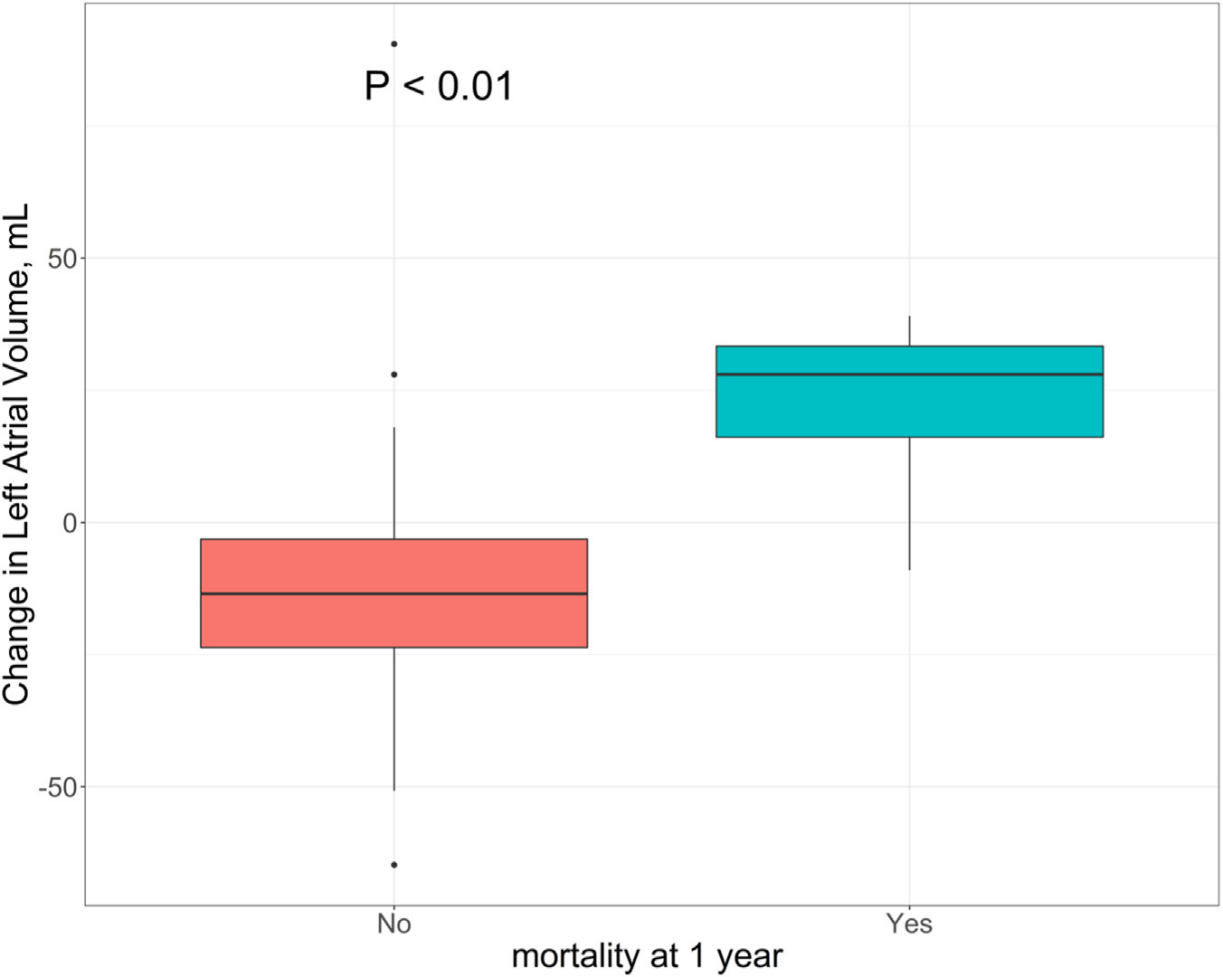
Change in left atrial volume by mortality at 1 year in patients undergoing transcatheter mitral valve-in-valve implantation.

However, the *change* in LAV was associated with 1-year all-cause mortality (OR 1.06; CI 1.01-1.12; *p* = 0.02) (Table 4, Model 1, Figure 1). When adjusted for small differences in postoperative examination time, given that data were sourced from the TVT registry where there is an allowed tolerance window, these findings do not significantly change (Table 4, Model 2).

**Table 4:** Association of change in pre and postprocedural echo parameters and 1-year mortality of 57 patients undergoing transcatheter mitral valve-in-valve implantation

Change in echo parameters	Model 1∗		Model 2∗	
	Hazard ratio (95% CI)	*p*-value	Hazard ratio (95% CI)	*p* value
LVEF, %	0.97 (0.87 - 1.08)	0.59	0.97 (0.87 - 1.08)	0.54
LVESD, cm	2.04 (0.27 - 15.28)	0.49	2.05 (0.27 - 15.48)	0.49
LVEDD, cm	0.54 (0.08 - 3.93)	0.55	0.54 (0.08 - 3.93)	0.55
LVGLS, %	1.09 (0.78 - 1.51)	0.61	1.11 (0.77 - 1.60)	0.56
LAV, ml	1.06 (1.01 - 1.12)	0.023	1.07 (1.01 - 1.14)	0.016
PALS, %	0.97 (0.82 - 1.16)	0.78	0.98 (0.82 - 1.17)	0.79
CS, %	0.85 (0.63 - 1.14)	0.28	0.85 (0.63 - 1.15)	0.29
PACS, %	1.01 (0.79 - 1.29)	0.92	1.01 (0.80 - 1.29)	0.91
RVFAC, %	0.98 (0.88 - 1.10)	0.79	0.98 (0.88 - 1.10)	0.78
TAPSE, cm	1.00 (0.78 - 1.26)	0.97	1.00 (0.78 - 1.27)	0.97
S’, cm/s	0.81 (0.48 - 1.37)	0.44	0.79 (0.45 - 1.39)	0.42
RVFWS, %	0.92 (0.71 - 1.18)	0.52	0.91 (0.70 - 1.17)	0.46
RVSP, mmHg	0.99 (0.92 - 1.06)	0.75	0.99 (0.92 - 1.06)	0.75
MV gradient, mmHg	1.34 (0.96 - 1.86)	0.08	1.46 (0.96 - 2.22)	0.08
Heart rate, bpm	1.02 (0.94 - 1.12)	0.58	1.03 (0.94 - 1.13)	0.51

Abbreviations: CI, confidence interval; CS, conduit strain; LAV, left atrial volume; LVEF, left ventricular ejection fraction; LVEDD, left ventricular end-diastolic dimension; LVESD, left ventricular end-systolic dimension; LVGLS, left ventricular global longitudinal strain; MV, mitral valve; PACS, peak atrial contractile strain; PALS, peak atrial longitudinal strain; RVSP, right ventricular systolic pressure; RVFAC, right ventricular fractional area change; RVFWS, right ventricular free-wall strain; TAPSE, tricuspid annular plane systolic excursion.

∗ Model 1 describes a Cox regression model of 1-year mortality adjusting for age, gender and baseline echo. Model 2 further adjusts for length of time to follow-up echo in days.

A decrease in median (IQR) LAV was observed in patients without mortality at 1 year and an increase in LAV was observed in all patients with 1-year mortality postintervention (-13.5 [-23.8 to -2.7] ml vs. LAV 28.0 [7.6-35.2] ml, *p* = <0.01) (Figure 1).

### Early cardiac remodeling in patients with atrial fibrillation/flutter following transcatheter MViV intervention

In a subgroup analysis conducted to evaluate differences in reverse remodeling in patients with atrial fibrillation/flutter compared to those without, 40 patients (65%) who underwent MViV intervention for failed mitral bioprosthetic valves had a history of atrial fibrillation/flutter.

No significant difference in baseline LAV (*p* = 0.80) or postprocedural LAV (*p* = 0.55) was observed in patients with atrial fibrillation/flutter compared to those without (Table 5).

**Table 5:** Baseline and Postprocedure echocardiographic characteristics of 62 patients undergoing transcatheter mitral valve-in-valve implantation between patients with and without atrial fibrillation/atrial flutter

Echo parameters, mean (SD) except as indicated	Baseline				Postprocedural			
	N	Atrial fibrillation/Atrial flutter			N	Atrial fibrillation/atrial flutter		
		Yes (n = 35)	No (n = 27)	*p* value∗		Yes (n = 35)	No (n = 27)	*p* value∗
LVEF, %	62	58.7 ± 10.6	58.4 ± 10.4	0.93	56	60.1 ± 9.7	59.1 ± 14.2	0.76
LVESD, cm	62	2.9 ± 0.8	3.1 ± 1.0	0.58	56	2.9 ± 0.8	3.2 ± 1.1	0.39
LVEDD, cm	62	4.5 ± 0.8	4.5 ± 1.0	0.85	56	4.4 ± 0.8	4.4 ± 1.0	0.99
LVGLS, %	60	-16.6 ± 4.6	-15.2 ± 4.8	0.24	55	-15.1 ± 3.9	-15.2 ± 5.6	0.95
LAV, ml, median (IQR)	62	106.6 (80.1-131.0)	95.0 (83.0-131.8)	0.80	56	97.0 (75.8-124.0)	93.2 (73.1-109.0)	0.55
PALS, %, median (IQR)	54	9.7 (6.2-14.4)	14.2 (10.1-19.5)	0.026	53	11.2 (9.0-15.3)	15.3 (10.7-20.7)	0.14
CS, %	54	8.3 ± 5.8	10.7 ± 5.5	0.13	53	9.9 ± 4.2	11.7 ± 5.3	0.17
PACS, %, median (IQR)	54	2.1 (1.0-5.2)	2.8 (1.0-8.2)	0.27	53	1.9 (0.9-3.3)	2.5 (1.7-7.3)	0.17
RVFAC, %	55	22.2 ± 9.9	18.6 ± 9.0	0.17	56	26.4 ± 9.1	27.3 ± 10.0	0.74
TAPSE, cm, median (IQR)	57	1.5 (1.1-1.7)	1.6 (1.2-1.7)	0.63	54	1.5 (1.2-1.9)	1.7 (1.5-1.9)	0.18
S’, cm/s	51	8.5 ± 1.9	9.2 ± 2.7	0.28	56	8.4 ± 2.6	8.5 ± 2.0	0.96
RVFWS, %	54	12.0 ± 4.3	10.4 ± 4.5	0.17	54	14.1 ± 5.1	14.0 ± 4.8	0.91
MV gradient, mmHg	61	12.5 ± 4.8	14.1 ± 5.0	0.21	55	51.9 ± 21.7	45.9 ± 17.3	0.26
RVSP, mmHg	61	61.4 ± 16.7	58.9 ± 17.3	0.58	56	7.7 ± 2.9	7.5 ± 3.1	0.80
Heart rate, bpm, median (IQR)	61	70.0 (61.0-83.0)	73.5 (61.0-85.0)	0.76	56	70.0 (66.0-82.0)	71.0 (60.0-83.0)	0.80

Abbreviations: CS, conduit strain; LAV, left atrial volume; LVEF, left ventricular ejection fraction; LVEDD, left ventricular end-diastolic dimension; LVESD, left ventricular end-systolic dimension; LVGLS, left ventricular global longitudinal strain; MV, mitral valve; PACS, peak atrial contractile strain; PALS, peak atrial longitudinal strain; RVSP, right ventricular systolic pressure; RVFAC, right ventricular fractional area change; RVFWS, right ventricular free-wall strain; TAPSE, tricuspid annular plane systolic excursion.

∗ Two sample *t* test, or Wilcoxon test as appropriate.

Although patients with atrial fibrillation/flutter had significantly lower median PALS compared to those without (9.7 vs. 14.2%) (*p* = 0.026), and a small but insignificant difference was noted between groups at follow-up (11.2 vs. 15.3%) (*p* = 0.14) (Table 5).

We also observed no significant difference in left ventricular chamber dimensions and function and right ventricular function parameters pre and postprocedurally in patients with or without atrial fibrillation/flutter (Table 5).

### Impact of MViV Intervention on NYHA Classification Improvement

**Figure 2:**
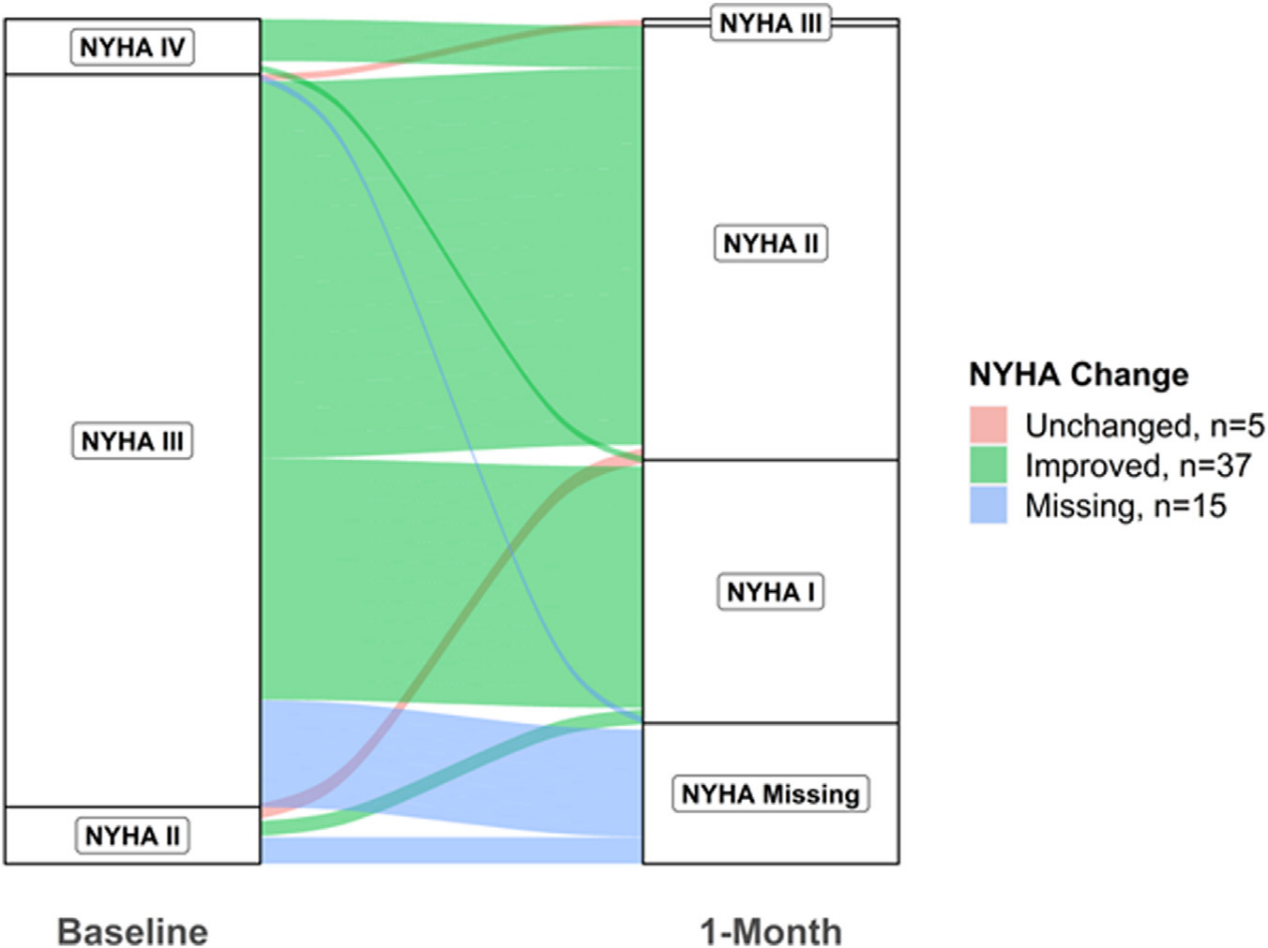
New York Heart Association (NYHA) functional class at baseline and 1-month among 57 patients undergoing transcatheter mitral valve-in-valve implantation.

Among the 57 patients in whom LAVs were measured pre and post MViV intervention, 37 (65%) patients had improvement in NYHA symptom classification at 1-month follow-up visit (Figure 2).

## Discussion

The results of this retrospective study of patients who underwent transcatheter MViV intervention for failed bioprosthetic MV confirm the following main findings:

(1) Early structural reverse remodeling of the left atrium as assessed by LAV occurs in patients following MViV intervention.
(2) Improvement of left atrial CS following MViV also occurs.
(3) Change in left atrial chamber remodeling is associated with all-cause mortality at 1 ​year.
(4) Early RV function improvement as assessed by FAC, S′, TAPSE, and strain occurs in patients following MViV.
(5) No significant left ventricular remodeling was observed in our study following MViV intervention.
(6) Improvement in heart failure symptoms occurs early following MViV intervention which is consistent with findings noted in larger registry data reports.^5^^,^^6^

Bioprosthetic valve stenosis and regurgitation is often caused by structural deterioration of the valve with associated leaflet thickening and calcification, pannus, or thrombus.

Severe prosthetic stenosis and regurgitation is often accompanied by secondary findings of left atrial dilatation due to increased chamber pressure and volume leading to downstream effects of elevated pulmonary capillary wedge pressures, and elevated right sided pressures that contribute to the development of heart failure symptoms. In this setting, echocardiography plays an important role in evaluating the mechanism and quantifying the severity of valvular dysfunction as described by the American Society of Echocardiography.^23^

Using echocardiography, our study provides, for the first time, data on cardiac structural and functional remodeling after MViV intervention which may offer more insight into the mechanisms driving the clinical improvements experienced by these patients and offer some understanding into their prognostication.

Our first finding demonstrating a reduction in LAV after MViV is consistent with well-documented findings after cardiac surgery and transcatheter intervention in native valves, where early LA reverse modeling resulted in decreased LA dimensions after MV repair.^24^, ^25^, ^26^ However, changes in LA contractility and function after MV intervention have not been entirely consistent; some studies have shown improvement in various indices while some have not shown beneficial changes.^27^, ^28^, ^29^ Our study showed that the improvement in atrial function by strain in the MViV cohort was due to improvements in contractility during the passive filling phase.

After MV surgery, Orde et al showed significant early RV dysfunction, likely due to a variety of factors including perioperative hypoperfusion, hypothermia and pericardial opening. In the transcatheter population however, a select group of patients can show acute improvement in RV function after MV repair.^30^ We show that even after MViV, there is improvement in right ventricular function as captured by FAC, S′, TAPSE, and strain in our cohort, likely due to improvement in right ventricular systolic pressure and right-sided remodeling.

In cardiac surgery, left atrial enlargement and left atrial strain have been shown to have significant prognostic implications in patients with severe mitral regurgitation undergoing MV surgery with baseline LAV and left atrial global longitudinal strain noted as independent predictors of postoperative cardiovascular events.^10^^,^^31^ Similarly, a study by Ledwoch et al^32^ investigating transcatheter mitral interventions in native valves identified changes in atrial function predict mortality and long-term outcomes. Our study showed no association between baseline LAV or LA strain with 1-year all-cause mortality in patients with failed bioprosthetic valves undergoing transcatheter MViV intervention. Instead, change in LAV was associated with 1-year all-cause mortality. Further research identifying the factors associated with changes in atrial volume and reverse remodeling as MViV interventions increase in volume across the country may provide opportunities to guide clinical decision-making with regards to patient selection and optimal timing for intervention to improve outcomes.

Notably, we observed through subgroup analysis lower baseline PALS in patients with atrial fibrillation/flutter compared to those without undergoing MViV intervention and a persistently lower but insignificant postprocedural PALS in the atrial fibrillation group.

These findings are consistent with a previously published pilot study by Albini et al. studying interventions in native valves.^33^ We hypothesize that significant baseline atrial cardiopathy is reflected in lower preprocedural PALS which could have an impact on reverse remodeling in atrial fibrillation patients after MViV but are limited by our small sample size.

This is particularly pertinent in the MViV cohort, who presumably have been experiencing atrial fibrillation and associated fibrosis for a longer period and are more likely to have undergone irreversible deleterious remodeling. Ultimately, we demonstrate that echocardiography provides a readily available tool for the assessment of prosthetic valve dysfunction and the evaluation of early structural and functional remodeling in patients who have undergone MViV intervention. We describe, for the first time, early left atrial and ventricular remodeling after MViV intervention, which may serve as a useful tool in predicting the risk of poor outcomes; however, further investigation into longitudinal changes and their long-term prognostic implication is needed.

### Study limitations

The limitations to this study are mostly derived from its retrospective, single-center nature with a relatively small sample size and particularly small number of deaths. Additionally, there was some missing echo data due to postprocedural image degradation related to prosthetic related artifacts. However, this study is the first and largest report to date exploring cardiac remodeling following MViV intervention in patients with bioprosthetic MV failure and its impact on outcomes.

## Conclusion

Following MViV intervention, there is significant early improvement in heart failure symptoms. Early reverse remodeling results in reduction in LAV, improvement in left atrial CS and improvement in right ventricle FAC, S′, TAPSE, and free wall longitudinal strain. Reverse remodeling following MViV intervention provides more insight into potential mechanisms driving clinical improvement and offer some understanding into prognosis of these patients.

## Ethics Statement

The research study was conducted in accordance with the Declaration of Helsinki and has adhered to the relevant ethical guidelines at Northwestern University.

## Funding

Funding for this paper was provided by Feis Family Philanthropic Support (Narang).

## Disclosure Statement

The authors report no conflict of interest.
