# Supplementary material for: Cardiac Structural and Functional Remodeling After Transcatheter Mitral Valve in Valve Implantation: Early Changes and Prognostic Significance
Source: Struct Heart. 2023 Dec 26;8(2):100264. doi: 10.1016/j.shj.2023.100264 (PMC10927451; doi:10.1016/j.shj.2023.100264)
Supplement: Table S1 [file mmc1.docx]

**Supplemental Table 1.** Characteristics of Echo Parameters by Valve Lesion Groups of 62 Patients Undergoing Transcatheter Mitral Valve-in-valve Implantation by valve lesion types

| **Echo parameters** | **Valve lesion group** | | | | **P-value^1^** |
| --- | --- | --- | --- | --- | --- |
|  | **N** | Stenosis  N=48 | Regurgitation  N=9 | Mixed  N=5 |  |
| **Baseline** |  |  |  |  |  |
| LVEF, % | 62 | 59.3 ± 9.1 | 58.0 ± 14.8 | 52.8 ± 14.2 | 0.42 |
| LVESD, cm | 62 | 2.9 ± 0.8 | 3.1 ± 1.4 | 3.4 ± 0.7 | 0.52 |
| LVEDD, cm | 62 | 4.4 ± 0.8 | 4.8 ± 1.3 | 5.0 ± 0.3 | 0.15 |
| LVGLS, % | 60 | -16.1 ± 4.7 | -16.5 ± 4.3 | -14.8 ± 6.2 | 0.81 |
| LAV, ml, median (IQR) | 62 | 104.0 (84.0-125.3) | 94.8 (79.5-165.6) | 141.0 (92.6-142.0) | 0.67 |
| PALS, %, median (IQR) | 54 | 11.5 (7.4-19.5) | 14.2 (9.7-14.9) | 10.6 (6.8-14.4) | 0.70 |
| CS, % | 54 | 8.7 ± 5.3 | 11.8 ± 8.1 | 9.6 ± 4.0 | 0.34 |
| RVFAC, % | 55 | 20.8 ± 9.4 | 19.5 ± 12.5 | 19.7 ± 6.3 | 0.92 |
| TAPSE, mm, median (IQR) | 57 | 14.0 (12.0-17.0) | 17.0 (15.0-21.0) | 14.0 (11.0-17.5) | 0.17 |
| S’, cm/s | 51 | 8.8 ± 2.2 | 9.2 ± 2.6 | 8.0 ± 2.8 | 0.66 |
| RVFWS, % | 54 | 11.3 ± 4.5 | 10.9 ± 5.2 | 12.0 ± 2.2 | 0.92 |
| RVSP, mmHg | 61 | 61.6 ± 16.7 | 54.8 ± 16.2 | 58.2 ± 21.3 | 0.53 |
| MV gradient, mmHg | 61 | 14.4 ± 4.6 | 7.6 ± 2.7 | 11.8 ± 4.6 | <0.01 |
| Heart rate, bpm, median (IQR) | 61 | 71 (61-85) | 62 (60-70) | 74.0 (70-82) | 0.33 |
| **Follow-up** |  |  |  |  |  |
| LVEF, % | 56 | 61.6 ± 9.7 | 53.0 ± 19.1 | 51.3 ± 10.8 | 0.057 |
| LVESD, cm | 56 | 2.9 ± 0.8 | 3.3 ± 1.4 | 3.6 ± 0.6 | 0.27 |
| LVEDD, cm | 56 | 4.4 ± 0.8 | 4.4 ± 1.5 | 5.1 ± 0.2 | 0.38 |
| LVGLS, % | 55 | -16.0 ± 4.5 | -12.9 ± 4.8 | -10.8 ± 3.2 | 0.031 |
| LAV, ml, median (IQR) | 56 | 95.5 (77.1-114.2) | 79.4 (62.5-138.0) | 99.6 (76.3-118.0) | 0.91 |
| PALS, %, median (IQR) | 53 | 13.6 (10.7-19.5) | 11.8 (7.8-16.9) | 9.5 (7.9-11.6) | 0.17 |
| CS, % | 53 | 11.4 ± 5.1 | 8.4 ± 3.5 | 9.0 ± 2.3 | 0.21 |
| RVFAC, % | 56 | 26.2 ± 8.9 | 30.2 ± 12.8 | 26.7 ± 8.7 | 0.55 |
| TAPSE, mm, median (IQR) | 54 | 16.0 (13.0-19.0) | 16.0 (13.0-18.0) | 17.0 (15.0-22.0) | 0.75 |
| S’, cm/s | 56 | 8.6 ± 2.1 | 7.9 ± 2.3 | 8.3 ± 4.6 | 0.73 |
| RVFWS, % | 54 | 14.1 ± 4.8 | 14.7 ± 5.8 | 12.6 ± 6.5 | 0.80 |
| RVSP, mmHg | 55 | 50.3 ± 20.9 | 44.4 ± 18.8 | 45.0 ± 9.0 | 0.70 |
| MV gradient, mmHg | 56 | 7.7 ± 2.9 | 5.7 ± 1.0 | 10.0 ± 4.7 | 0.050 |
| Heart rate, bmp, median (IQR) | 56 | 71.5 (66.0-82.5) | 66.5 (56.5-71.5) | 78.0 (57.5-93.5) | 0.37 |

^1^ ANOVA test or Kruskal–Wallis as appropriate.
