# Supplementary material for: Cardiac Structural and Functional Remodeling After Transcatheter Mitral Valve in Valve Implantation: Early Changes and Prognostic Significance
Source: Struct Heart. 2023 Dec 26;8(2):100264. doi: 10.1016/j.shj.2023.100264 (PMC10927451; doi:10.1016/j.shj.2023.100264)
Supplement: Table S2 [file mmc2.docx]

**Supplemental Table 2.** Association of baseline echo parameters and post-op residual MR of 62 Patients Undergoing Transcatheter Mitral Valve-in-valve Implantation

| **Echo parameters** | **Post-op residual MR** | | | | **P-value^1^** |
| --- | --- | --- | --- | --- | --- |
|  | **N** | None  N=20 | Trace/Trivial  N=31 | Mild  N=9 |  |
| LVEF, % | 62 | 54.5 ± 12.5 | 59.9 ± 8.4 | 62.8 ± 8.4 | 0.07 |
| LVESD, cm | 62 | 3.4 ± 1.2 | 2.8 ± 0.7 | 2.7 ± 0.5 | 0.07 |
| LVEDD, cm | 62 | 4.8 ± 1.1 | 4.3 ± 0.7 | 4.1 ± 0.6 | 0.058 |
| LVGLS, % | 60 | -15.2 ± 4.7 | -16.5 ± 4.1 | -16.6 ± 6.4 | 0.60 |
| LAV, ml, median (IQR) | 62 | 118.8 (83.8-137.3) | 99.0 (81.0-122.0) | 94.5 (78.5-114.0) | 0.27 |
| PALS, %, median (IQR) | 54 | 11.1 (8.3-12.6) | 11.5 (7.4-20.0) | 18.4 (7.6-19.5) | 0.43 |
| CS, % | 54 | 7.3 ± 3.4 | 10.0 ± 6.1 | 10.8 ± 8.7 | 0.26 |
| RVFAC, % | 55 | 17.4 ± 8.7 | 21.6 ± 8.8 | 24.4 ± 14.3 | 0.19 |
| TAPSE, mm, median (IQR) | 57 | 15.0 (12.0-16.0) | 15.0 (12.0-21.0) | 12.5 (10.5-17.0) | 0.29 |
| S’, cm/s | 51 | 8.6 ± 2.2 | 9.3 ± 2.2 | 7.8 ± 3.1 | 0.30 |
| RVFWS, % | 54 | 9.5 ± 3.9 | 11.8 ± 4.8 | 13.7 ± 2.9 | 0.06 |
| RVSP, mmHg | 61 | 59.1 ± 15.0 | 60.9 ± 18.9 | 61.6 ± 12.4 | 0.91 |
| MV gradient, mmHg | 61 | 12.7 ± 5.1 | 13.0 ± 5.0 | 15.3 ± 4.8 | 0.40 |

^1^ ANOVA test or Kruskal–Wallis as appropriate.
