# Supplementary material for: Cardiac Structural and Functional Remodeling After Transcatheter Mitral Valve in Valve Implantation: Early Changes and Prognostic Significance
Source: Struct Heart. 2023 Dec 26;8(2):100264. doi: 10.1016/j.shj.2023.100264 (PMC10927451; doi:10.1016/j.shj.2023.100264)
Supplement: Table S3 [file mmc3.docx]

**Supplemental Table 3**. Association of 1-year mortality and conduit strain adjusted for LV function

| **Characteristic** | **Model 1^*^** | |  | **Model 2*** | |
| --- | --- | --- | --- | --- | --- |
|  | **Hazard ratio (95% CI)** | **P-value^1^** |  | **Hazard ratio (95% CI)** | **P-value^1^** |
| Baseline CS, % | 0.90 (0.72 - 1.12) | 0.35 |  | 0.91 (0.74 - 1.11) | 0.34 |
| Change in CS | 0.85 (0.63 - 1.14) | 0.28 |  | 0.87 (0.65 – 1.16) | 0.34 |

*Model 1 for baseline CS: Mortality = Baseline CS + Age + Gender;

Model 2 for baseline CS: Mortality = Baseline CS + Age + Gender + Baseline LVEF + Baseline LVGLS;

Model 1 for Change in CS: Mortality = Change in CS + Age + Gender + Baseline CS;

Model 2 for baseline CS: Mortality = Change in CS + Age + Gender + Baseline CS + Change in LVEF + Change in LVGLS
